# PUF60 Promotes Chemoresistance Through Drug Efflux and Reducing Apoptosis in Gastric Cancer

**DOI:** 10.7150/ijms.102976

**Published:** 2025-01-01

**Authors:** Qianhui Liu, Yingqiu Song, Jing Su, Shangbin Yang, Qinghai Lian, Tiantian Wang, Hongbo Wei, Jiafeng Fang

**Affiliations:** 1 Department of Gastrointestinal Surgery, the Third Affiliated Hospital of Sun Yat-sen University, Guangzhou, China.; 2 Department of Nursing, the Third Affiliated Hospital of Sun Yat-sen University, Guangzhou, China.; 3 Department of Cell-Gene Therapy Translational Medicine Research Center, the Third Affiliated Hospital of Sun Yat-Sen University, Guangzhou, China.; 4 Department of Medical Oncology, the Third Affiliated Hospital of Sun Yat-sen University, Guangzhou, China.

**Keywords:** Gastric cancer, PUF60, Chemoresistance, Prognostic biomarkers

## Abstract

**Background:** Chemotherapy resistance is a great challenge in the treatment of gastric cancer (GC), so it is urgent to explore the prognostic markers of chemoresistance. PUF60 (Poly (U)-binding splicing factor 60) is a nucleic acid-binding protein that has been shown to regulate transcription and link to tumorigenesis in various cancers. However, its biological role and function in chemotherapy resistance of GC is unclear.

**Methods:** The expression and prognostic value of PUF60 in GC chemotherapy-resistant patients were analyzed by databases and K-M Plotter. The functional effect of PUF60 on chemoresistance in GC was studied by by RNA interference, CCK8 test, colony formation test and apoptosis detection. Moreover, further validation and mechanism exploration were conducted in clinical samples.

**Results:** PUF60 was highly expressed in both GC and chemoresistant tissues, and was positively correlated with poor prognosis in GC patients treated with 5-fluorouracil (5-FU). In addition, the knockdown of PUF60 significantly reduced the proliferation of human gastric cancer cells and increased sensitivity to chemotherapy drugs, such as 5-FU and cisplatin (CDDP). Mechanistically, PUF60 enhances chemotherapy resistance in gastric cancer (GC) cells by actively excluding chemotherapy drugs via the recombinant ATP Binding Cassette Transporter A1 (ABCA1) and ATP Binding Cassette Subfamily C Member 1 (ABCC1). This process further affects the cell cycle, reduces cell apoptosis, and ultimately promotes resistance to chemotherapy in GC.

**Conclusion:** PUF60 promotes chemoresistance in GC, resulting in poor prognosis of GC patients treated with 5-FU, and providing a new idea for overcoming the chemoresistance in GC.

## Introduction

Globally, gastric cancer (GC) is the fifth most common cancer and ranks third in terms of cancer-related death^1^, ^2^. Chemotherapy remains the cornerstone of treatment for advanced GC^3^. However, the emergence of chemoresistance has become a major obstacle in the clinical treatment of GC^4^. Therefore, it is urgent to identify the potential biomarkers and relevant mechanisms directly related to chemotherapy resistance in GC.

Poly(U) binding splicing factor 60 (PUF60), also known as FUSE-binding Protein-interacting repressor or Ro-binding protein 1(Ro-bp1), is a nucleic acid-binding protein. PUF60 directly binds to RNA and DNA and is involved in multiple nuclear processes, such as pre-mRNA splicing^5^ and transcriptional regulatory^6^. The relative abundance of PUF60 influences the choice of alternative splice sites^7^. Recent studies have revealed that PUF60 promotes the development of a variety of cancers, containing colon cancer^8^, ^9^, ovarian cancer^10^, hepatocellular carcinoma^11^, bladder cancer^12^, non-small cell lung cancer^13^, breast cancer^14^, ^15^, esophageal cancer^16^ and renal cell carcinoma^17^. Some studies have suggested that PUF60 may be a novel potential driver through integrated copy number and expression analysis^18^. In addition, abnormal expression or mutation of PUF60 has been widely reported in congenital diseases associated with intellectual disability, cardiac defects, and short stature^19^-^22^. However, few existing studies have directly explored the association of PUF60 with GC chemoresistance.

Cytotoxic drugs such as 5-fluorouracil and cisplatin are widely used in GC chemotherapy and induce apoptosis in cancer cells mainly through the intrinsic apoptosis pathway^23^, ^24^. However, the high heterogeneity of GC and the complex nature of its chemoresistance mechanisms, including reduced intracellular drug concentration, altered drug targets, and disordered cell survival and death pathways^25^, ^26^, attribute to major challenges to achieve the prospective effect.

In this study, we innovatively investigated the role of PUF-60 in GC chemoresistance and found that in GC patients treated with 5-FU, PUF60 was significantly up-regulated after resistance and was confirmed in TCGA, GC cell lines and clinical tissues. In addition, the high expression of PUF60 led to poor prognosis of GC patients treated with 5-FU chemotherapy. Moreover, it was found that PUF60 promoted chemoresistance in GC through drug efflux and reduced apoptosis. Taking together, our study indicates that PUF60 might be a novel therapeutic target for overcoming GC chemoresistance.

## Material and Methods

### Data acquisition and functional enrichment analysis

The expression level of PUF60 was analyzed using the Cancer Genome Atlas (TCGA) database, the CCLE database (https://portals.broadinstitute.org/ccle/) and the Gene Expression Omnibus (GEO, https://www.ncbi.nlm.nih.gov/geo/). Survival rate analyzed by a Kaplan-Meier analysis were referenced from an online database-The Kaplan Meier plotter (https://kmplot.com/analysis/)^27^. The online STRING database (https://string-db.org/, V11.0)^28^ and Cytoscape (https://cytoscape.org/)^29^ is used to analyze all publicly available sources of information and predict protein-protein interactions in the organism. The entrez ID was converted to gene symbols using the Hs.eg.db " (v3.10.0) R package. Functional annotation used KEGG analysis using the "Cluster Analyzer" (v3.14.3) R package.

### Clinical samples

Clinical GC tissues (January 2013 to December 2021) receiving neoadjuvant chemotherapy at the Third Affiliated Hospital of Sun Yat-sen University were included in this study. Written informed consent was obtained, and the experiments related to human samples were approved by the Ethics Committee of the Third Affiliated Hospital of Sun Yat-sen University ([2022] 02-169-01) to obtain fresh-frozen normal stomach and tumor tissues. Patient Inclusion Criteria: 1) Pathologically confirmed diagnosis of gastric cancer; 2) Admitted to the Gastrointestinal Surgery Department of the Third Affiliated Hospital of Sun Yat-sen University between 2013 and 2021; 3) Patients receiving a neoadjuvant treatment regimen based on 5-FU and/or immunotherapy; 4) Underwent surgical treatment and had specimens retained in the clinical sample resource bank; 5) Available and traceable CT results. Exclusion Criteria: 1) Absence of a pathologically confirmed diagnosis of gastric cancer; 2) Did not undergo surgical treatment; 3) Patients who did not receive chemotherapy based on 5-FU; 4) Inability to assess therapeutic efficacy based on follow-up CT results. The effect of 5-FU chemotherapy was evaluated by RECIST 1.1, including CR (complete response), PR (partial response), SD (stable disease), and PD (progressive disease). The patients were divided into two groups: the sensitive group (CR, PR) and the non-sensitive group (SD, PD).

### Cell culture and reagents

Human GC cell lines HGC-27, AGS, MGC-803, MKN1, MKN45, YCC-2, human normal gastric cell line GSE-1, human embryonic kidney 293T (HEK293T) cells were all preserved in the Third Affiliated Hospital of Sun Yat-sen University. GSE-1, HGC-27, AGS, MGC-803, MKN1, MKN45, YCC-2 were cultured in RPMI 1640 containing 10% fetal bovine serum (FBS) and 1% penicillin/streptomycin (P/S). HEK293T were cultured in Dulbecco's modified Eagle's medium containing 10% fetal bovine serum (FBS). All cells were incubated at 37 ℃ in a humidified atmosphere containing 5% CO_2_.

### RNA isolation and quantitative real-time PCR

Total cellular RNA was extracted using Trizol reagent (Takara). PrimeScript RT-PCR kit (Takara) was used to perform the RT according to the protocol. Real-time PCR was used to determine the mRNA expression on a 7500 real-time PCR system (Applied Biosystems) according to the manual. In PCR experiments, each sample undergoes at least 3 biological replicates and technical replicates. Data were normalized to 18S RNA expression and represented as the average of three repeated experiments. Primer sequences used for correlated genes detection were shown in Supplementary Table 1.

### Western blotting

Total cellular protein and nuclear-cytosol protein were extracted using a total protein extraction buffer (Beyotime, China). Cell lysates were separated by SDS-PAGE. Then transfer the proteins from the gel to the membrane followed by blocking in 1% BSA (Bovine Serum Albumin). Incubate the primary antibody at 4°C overnight. The next day, after incubating with the secondary antibody, develop the bands for visualization. Bound secondary antibodies were detected with the Odyssey imaging system (LI-COR Biosciences, Lincoln, NE). The primary antibodies used and their corresponding dilution concentrations are as below: PUF60(1:1500), β-actin (1:1000), GAPDH (1:2000), P53 (1:1000), Bcl-2 (1:1000), ABCA1 (1:1500) and ABCC1 (1:1000). Each experiment was conducted with 3 biological replicates and used for relevant statistical analysis and graphing.

### Immunofluorescence (IF) and Immunohistochemistry (IHC)

Immunofluorescence was used to detect in five pairs of tumor tissues and adjacent normal tissues from gastric cancer patients. In immunohistochemical experiments, clinical sample tissues were classified into three groups based on the efficacy of chemotherapy: PD, SD and PR, to detect the expression levels of PUF60. Acquired clinical specimens were processed for formalin fixation, tissue cut into 5 µm sections for IHC staining. Briefly, the slides were dewaxed with xylene and ethanol, and the antigen was repaired with EDTA buffer (pH 8.0) in a microwave oven. After incubation with 5% goat serum diluted in TBS buffer containing 1% Tween-20, each with primary antibodies PUF60 (1:400) at 4℃ overnight and the following day with the corresponding species for 1 h at 37 degrees, DAB development of these immune cell markers was 3 min. To analyze the expression levels of PUF60 in different gastric tissue samples, we performed positive area ratio statistics on stained sections using Fiji software to compare the differences between the three groups. The most typical image was selected for presentation.

### Plasmid transfection

The sequences of the short hairpin (sh)RNAs targeting PUF60 were sh-1, 5'-CGATGACATCAAGAGCGTGTT-3', sh-2, 5'-TGCAGAAATCATTGTCAAGAT-3' and sh-3, 5'-CGTCCCAAGATGCTGTGTCTT-3'. The shRNA plasmids and control plasmid were purchased from GenePharma (Shanghai, China). All these plasmids were packaged into virus particles using HEK 293T cells and the viral titers were determined. Then the target cells were infected with 1× 10^8^ lentivirus-transducing units with 10 µg/mL polybrene (Sigma-Aldrich, St. Louis, MO, USA). The infected cells were then screened with 1 µg/mL puromycin after 72 h. The efficiency of the knockdown or overexpression was verified by western blotting.

### CCK8 experiments

Cell proliferation was measured with the CCK-8 reagent. Seeding 8000 cells / well in 96-well culture plates and add them 100 µL medium containing 10% FBS. Then, the adherent cells were treated with CDDP, 5-FU of the corresponding concentration gradient. The concentration gradients set for CDDP are: 0, 3, 5, 10, 25, 50, 100, and 300 μM; and for 5-FU, the concentration gradients are: 0, 3, 5, 10, 25, 50, 100, 300 μM. After 48 or 72h of treatment, 10 µL CCK-8 reagent was added and incubated at 37℃ and 5% CO_2_ for another 1 hour. Absorbance measured at 450 nm was used to plot the cell growth curves. Each of the above experiments was performed with 3 biological replicates.

### Colony formation test

The human gastric cancer cell line HGC-27, including control and knockdown groups (sh1, sh2), were seeded in 12-well plates at a density of 500 cells per well, treated or untreated with appropriate concentrations of 5-FU or CDDP and cultured continuously for 10 days. Based on the CCK8 results, which showed different IC50 values of cells to the drugs, the treatment concentrations of 5-FU for each group were set at 0, 0.5, 1, and 1.5 μM; and the treatment concentrations of CDDP were set at 0, 0.25, 0.5, and 1 μM. Colonies were then stained with 1% crystal violet and the number of clones was counted, and individual colonies were photographed under the microscope. Each of the experiments was performed with 3 biological replicates. And plot graphs based on the average number of colony formations in each group.

### Cell apoptosis assay

Cell apoptosis assay was performed using an Annexin V/7-AAD apoptosis kit (BD Biosciences, Franklin Lakes, NJ, USA) following the manufacturer's protocol. The human gastric cancer cell lines HGC-27, including control and knockdown groups (sh1, sh2), were seeded in 6-well plates at a density of 2 * 10^5^ cells per well, and cells were treated with 30 uM 5-FU, 20uM CDDP on the following day. After 48 hours, the cells were detached with 0.25% trypsin (without ethylenediaminetetraacetic acid), washed, resuspended with binding buffer and stained. Then cells were determined by flow cytometry using BD FACS Calibur (BD Biosciences). Each experiment was conducted with 3 biological replicates, and the results were subjected to statistical analysis.

### Statistical analysis

Statistical analysis was performed using GraphPad Prism (GraphPad, version 7). The statistics of the TCGA dataset were processed using R 3.6.3. The Wilcoxon rank-sum test and the Wilcoxon signed-rank test were used to compare the expression differences of PUF60 between gastric cancer and normal groups. Chi-square and t-test, and correlation analysis by Spearman's rank correlation. Correlation of PUF60 expression with categorical clinical variables in patients with OC was evaluated by χ2 test or Fisher's exact test. The student's t-test or one-way ANOVA was used for comparison between groups. All the experiments performed in this study were performed using three independent trials. A *P*-value of <0.05 was statistically significant. Data were presented as the mean±SD (*p < 0.05, **p < 0.01, *** p < 0.001, **** p < 0.0001).

## Results

### PUF60 Was the Core Upregulated Gene and Led to Poor Prognosis in GC Patients Treated with 5-FU-based Chemotherapy

**Figure 1:**
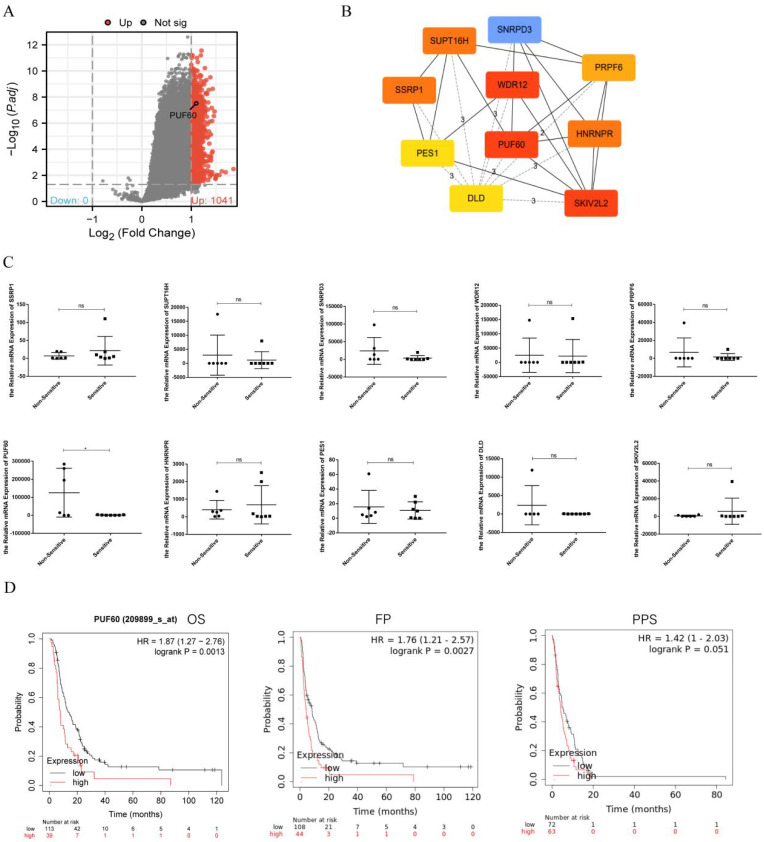
PUF60 expression was elevated and related to poor prognosis in GC Patients treated with 5-FU-based Chemotherapy. (A) Volcano plots of the differentially expressed genes (DEGs) screened by GEO2R in GSE14210. (B) The top 10 hub genes of above upregulated DEGs by MCC degree. (C) The mRNA expression level of 10 hub genes in the two groups. (D) Prognostic value of PUF60 in GC patients based on 5-FU chemotherapy by Kaplan-Meier plotter analysis. OS: overall survival, FP: first progression, PPS: post progression survival. According to the degree of chemotherapy response of the patients, they were divided into non-sensitive group (n=6) and sensitive group (n=7). The 13 GC patients here had paired tumor and normal tissues. ns, p≥ 0.05, *p < 0.05.

The GSE14210 dataset had gene expression data of both pretreatment and posttreatment endoscopic biopsy samples collected from GC patients treated with cisplatin and fluorouracil combination chemotherapy. Through GEO2R, totally 1041 different expression genes were found and shown by volcano plot (Figure 1A). The top 10 hub genes—PUF60, WDR12, SKIV2L2, HNRNPR, PRPF6, SNRPD3, SUPT16H, SSRP1, PES1, and DLD—were selected based on the MCC modules of STRING and Cytoscape (Figure 1B). These genes were then validated in GC patients undergoing chemotherapy. The patients were categorized into two groups: the sensitive group (CR, PR) and the non-sensitive group (SD, PD). The comparison of mRNA expression levels of 10 hub genes from the qPCR results showed that PUF60 expression was higher in the non-sensitive group, whereas the others had no difference between the two groups (Figure 1C). Therefore, PUF60 was considered to be the core gene associated with chemoresistance in GC. Then a survival analysis of PUF60 by Kaplan-Meier plotter was performed to evaluate its prognostic value for GC patients treated with 5-FU-based chemotherapy. As shown in Figure 1D, the higher PUF60 expression levels in GC patients showed a worse prognosis in GC patients treated with 5-FU based chemotherapy. Especially, the expression of PUF60 in GC patients treated with 5-FU based chemotherapy was negatively associated with overall survival (OS, HR = 1.87, p=0.0013) and first progression (FP, HR= 1.76, p=0.0027). Collectively, these results indicated that PUF60 may serve as a novel potential prognostic marker in GC patients with chemoresistance.

### The Expression Level of PUF60 Significantly Elevated in Gastric Cancer

**Figure 2:**
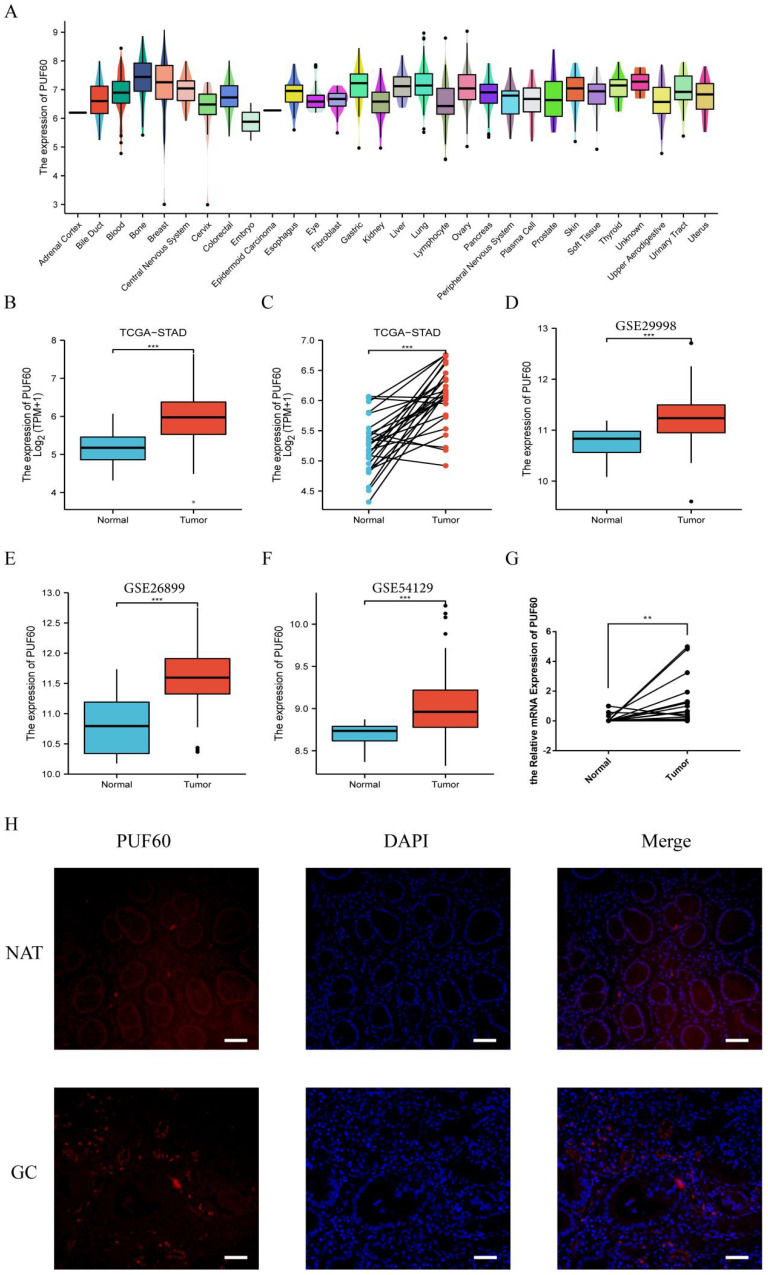
Expression level of PUF60 in GC by databases and experimental validation. (A) The expression level of PUF60 in pan-tumor cell lines analyzed by the CCLE database. (B-C) Expression level of PUF60 in unpaired or paired GC and normal tissues analyzed by TCGA database; (D-F) Expression level of PUF60 in GC and normal tissues analyzed by GEO database; (G) Comparison of the mRNA levels of PUF60 in paired GC samples. (H) Representative IF images of PUF60 from GC samples in gastric tumor (T) and adjacent normal tissues (NAT) by IF. **p < 0.01, ***p < 0.001.

A further exploration of PUF60 in GC were conducted. Firstly, CCLE database analysis showed that PUF60 was also higher in GC cell lines (Figure 2A). By TCGA analysis, the expression of PUF60 in normal tissues was significantly lower than that of unpaired or paired GC tissues (Figure 2B and C). Simultaneously, the same conclusion was validated in the GEO datasets, including GSE29998, GSE26899, GSE54129 (Figure 2D-F). Moreover, the expression of PUF60 in clinical samples was verified and the result revealed that the mRNA expression level of PUF60 was greatly higher in paired GC tissues (Figure 2G). Also, the IF experiments showed that the expression of PUF60 was significantly higher in GC tissues compared to adjacent normal tissues (Figure 2H). All the above results demonstrated that PUF60 was highly expressed in GC.

### PUF60 Was Closely Related to Chemoresistance in GC samples

**Figure 3:**
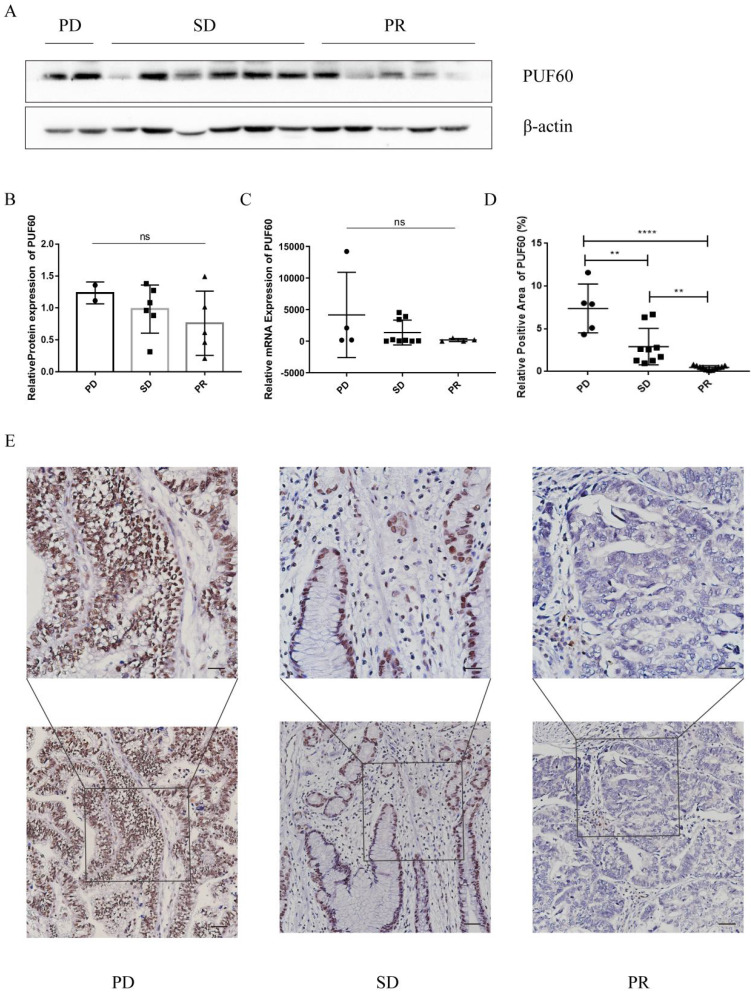
Expression level of PUF60 in different chemotherapy sensitivity groups. (A-B) The protein levels and corresponding bar graphs of PUF60 expression in GC tissues of the three groups. (C) The mRNA levels of PUF60 in GC tissues of the three groups; (D-E) Representative IHC images of PUF60 from GC samples and corresponding bar graphs. The groups are classified according to the degree of chemotherapy sensitivity, namely, PD: disease progression. SD: stable disease. PR: partial response. ns, p≥ 0.05, **p < 0.01, ****p < 0.0001.

GC patients were divided into three groups according to their chemotherapy sensitivity (PD, SD, or PR group). Then corresponding WB, qPCR and IHC experiments were conducted. Surprisingly, we found that the expression trend of PUF60 was negatively correlated with the chemotherapy sensitivity in GC patients, namely PD> SD> PR (Figure 3A-D). In especial, the results of IHC showed the significant difference among the three groups (Figure 3E), where PUF60 has the highest expression in PD group. Thus, it was preliminarily proved that PUF60 is closely correlated with chemoresistance in GC.

### PUF60 Promoted Chemoresistance of GC Cells *in vitro*

**Figure 4:**
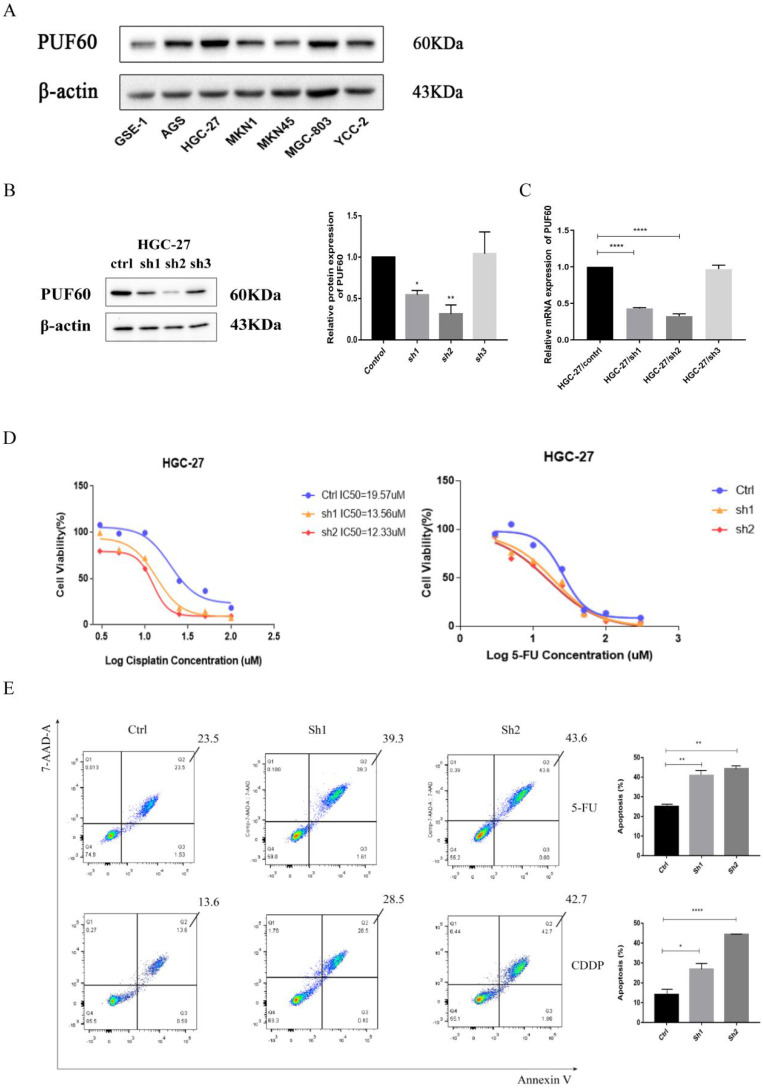
Knockdown of PUF60 in human gastric cancer cell line HGC-27 and its drug sensitivity to 5-FU and CDDP. (A) Expression level of PUF60 in different human gastric cancer cell lines. (B) Interference efficiency verification of PUF60 in HGC-27 cells (Ctrl, sh1, sh2). Left: western blot gels. Right: protein quantification, interference group values were compared with the control group. (C) The mRNA quantification in HGC-27; (D) Relative cell viability of HGC-27 cells expressing shNC and shPUF60 treated with different concentrations of CDDP and 5-FU by CCK 8 experiment. (E) Flow cytometry for detection of apoptosis by Annexin/7-AAD double staining in HGC-27 cells expressing shNC and shPUF60. Data are presented as the means±SEM. *p < 0.05, **p < 0.01, ****p < 0.0001.

The loss-of-function study in GC cells was conducted to elucidate the biological functions of PUF60 in GC with chemotherapy. As shown in Figure 4A, the WB results revealed that the expression of PUF60 was relatively higher in human GC cell lines than GES -1, especially HGC-27 cell line. Then RNA interference was applied to stably reduce the expression of PUF60 in GC cell lines. Even though we attempted PUF60 knockdown experiments multiple times across five cell lines, the results consistently showed stable knockdown of PUF60 in HGC-27 cells (Figure 4B-C), but not significantly in other cell lines like MKN45, MKN1, AGS and MGC803 (Supplementary Figure 1). Despite the regrettable inability to validate across multiple cell lines, considering our study's innovative discovery of the significant potential of PUF60 in GC chemoresistance, we continued further exploration and validation specifically in HGC-27 cells. As shown in the HGC-27 results of qPCR and WB, both group of sh1 and sh2 were successfully knocked out compared with the control group, and the sh2 group had the best knock-out efficiency (Figure 4B-C).

The significant upregulation of PUF60 in GC patients treated with 5-FU combined with CDDP in GSE14210 suggested that PUF60 promoted chemoresistance to both 5-FU and CDDP. HGC-27 cells were treated with 5-FU or CDDP for verification. The effect of PUF60 on the IC50 of GC cells was investigated through CCK-8 assays (Figure 4D). The results indicated that compared with the control group, the knockdown groups (sh1 and sh2) were more sensitive to chemotherapeutic drugs, especially after cisplatin treatment. The curves of CCK 8 shifted to the left with the decrease of PUF60 expression (Ctrl IC50=19.57uM, sh1 IC50=13.56uM, sh2 IC50=12.33uM). It showed that PUF60 was positively correlated with IC50 value of both 5-FU and CDDP. Additionally, the effects of PUF60 on GC cell apoptosis were explored by flow cytometry, where 20uM CDDP and 30 uM 5-FU were treated in HGC-27. With either 5-FU or CDDP treatment, the apoptosis rates of PUF60 knockdown groups were significantly higher than that in the control group (Figure 4E). In conclusion, it was proved that PUF60 promoted the inhibition of apoptosis of GC cells and enhanced the resistance of gastric cancer cells to chemotherapy drugs.

### PUF60 promoted proliferation and chemotherapeutic resistance of GC cells *in vitro*

**Figure 5:**
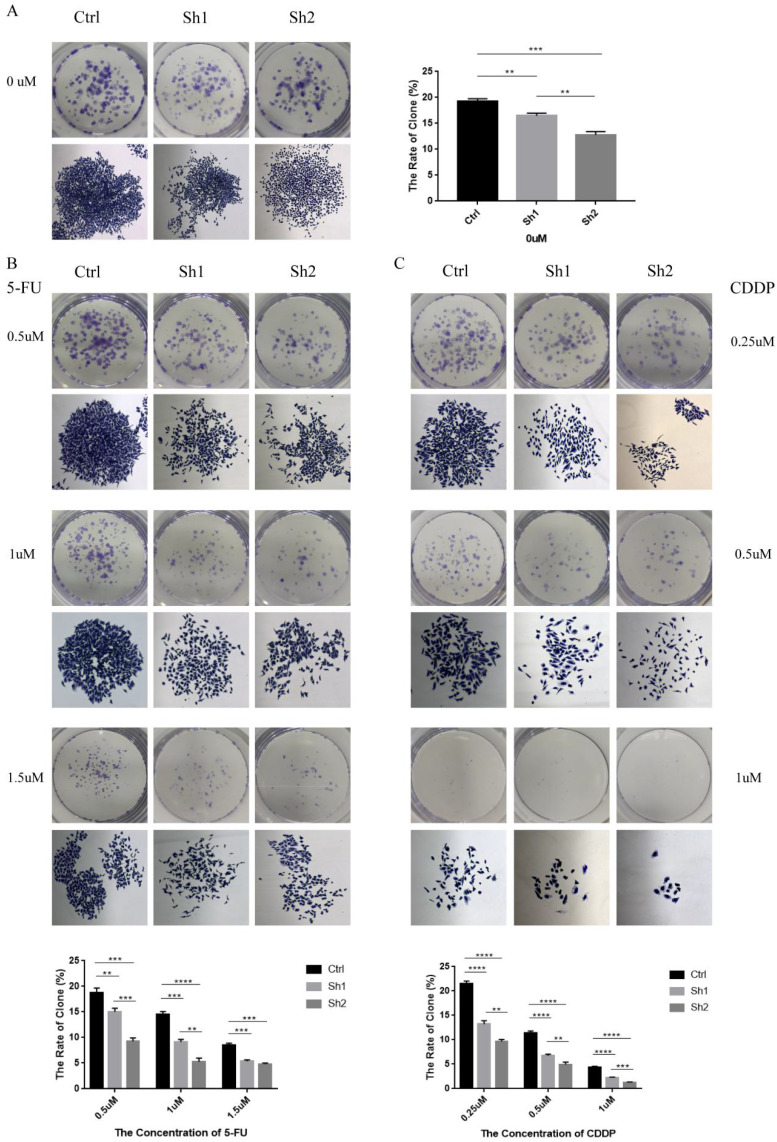
Low expression of PUF60 decreased the proliferative capacity and colony formation of HGC-27 cells expressing shNC (Ctrl) and shPUF60 (sh1, sh2). There are representative images of macroscopic and microscopic observation and relevant bar graphs for quantification. (A) Without drug treatment. (B) 5-FU treatment (0.5uM, 1uM, 1.5uM). (C) CDDP treatment (0.25uM, 0.5uM, 1 uM). Data are presented as the means±SEM. **p < 0.01, ***p < 0.001, ****p < 0.0001.

To explore the effect of PUF60 on proliferation and chemotherapy resistance of human GC cells, colony stimulation formation experiments were conducted. As shown in Figure 5A, the number of colony formation and cell growth status were significantly reduced in the knockdown group (sh1, sh2) compared with the negative control group (Ctrl). HGC-27 cells expressing shNC and shPUF60 were then stimulated with different concentrations of 5-FU (0.5uM, 1uM, 1.5uM) (Figure 5B). The result revealed that the sensitivity of the same cells to 5-FU was drug concentration-dependent. Moreover, there were fewer and smaller colonies with low PUF60 expression (sh2 <sh1 <ctrl). In other words, the expression level of PUF60 was positively correlated with colony formation even under 5-FU treatment. Additionally, different concentrations of CDDP (0.25uM, 0.5uM, 1 uM) were used (Figure 5C). The results also revealed that compared with the knockdown group, PUF60 significantly improved colony formation ability. In conclusion, PUF60 promoted chemotherapy resistance in GC.

### PUF60 May Promote Chemoresistance in GC Through Drug Efflux and Reducing Apoptosis

**Figure 6:**
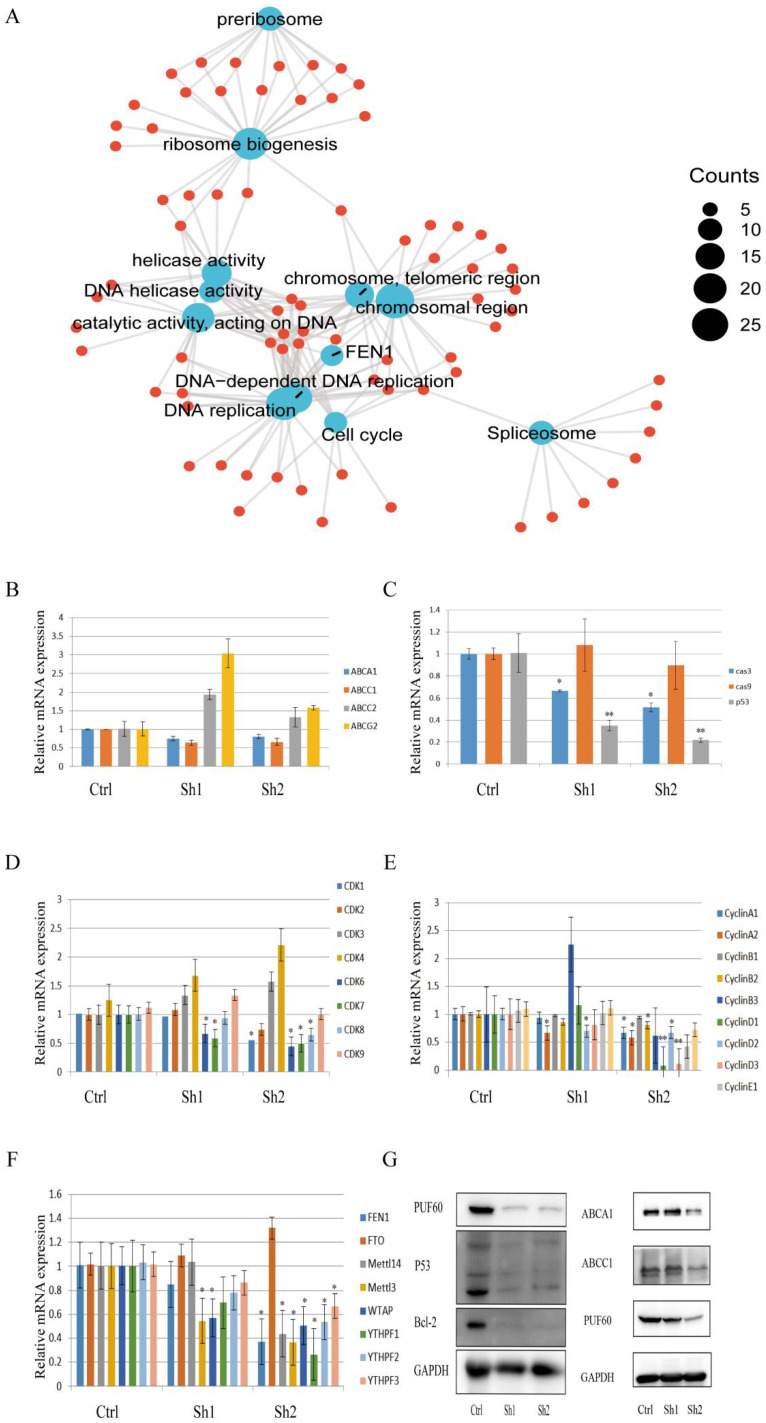
Mechanisms Underlying PUF60's Role in Enhancing Chemoresistance in GC. (A) Functional Enrichment Analysis: This panel presents the functional enrichment analysis of PUF60 in GC, suggesting its potential roles and interactions within the cellular context. (B) mRNA Expression Levels of ABC Protein Transporters: The expression levels of various ABC protein transporters (ABCA1, ABCC1, ABCC2, ABCG2) are shown. These data indicate how PUF60 may regulate the expression of these transporters, thereby affecting drug resistance. (C) mRNA Expression Levels of Apoptosis-Related Factors: The expression levels of apoptosis-related factors (caspase-3/cas3, caspase-9/cas9, p53) are presented. These factors are crucial for cancer cell death and their regulation by PUF60 may contribute to chemoresistance. (D-E) mRNA Expression Levels of Cell Cycle Factors: The expression levels of cell cycle factors, including all cyclin-dependent kinases and cyclin family members, are displayed. These data illuminate how PUF60 may disturb the normal cell cycle progression, thereby promoting chemoresistance. (F) mRNA Expression Levels of FEN 1 and Associated m6A Methylation Genes: The expression levels of FEN 1 and its associated m6A methylation genes (FTO, METTL14, METTL3, WTAP, YTHPF1, YTHPF2, YTHPF3) are shown. These data hint at a potential mechanism through which PUF60 may regulate DNA repair and replication processes, contributing to chemoresistance. Data are presented as the means±SEM. *p < 0.05, **p < 0.01.

To explore the underlying mechanism of PUF60 in chemoresistance of GC, functional enrichment analysis was firstly performed (Figure 6A) and the result showed that PUF60 played a major role in DNA unwinding, replication, spliceosome, ribosome and regulating the cell cycle. Also, the FEN1 gene was closely associated with PUF60, where FEN1 was a gene closely related to m6A methylation. Moreover, PUF60 promoted the chemoresistance of human GC cells to both 5-FU and CDDP, and multidrug resistance was often associated with ABC transporters. Therefore, the relevant qPCR test was performed. The results revealed that the mRNA level of ABCA1 and ABCC1 were elevated in the group with relatively high expression of PUF60 (Figure 6B), suggesting that PUF60 may improve drug resistance by promoting chemotherapeutic efflux through ABCA1 and ABCC1. Additionally, related genes were detected by qPCR based on the enrichment analysis. In terms of cell cycle and apoptosis, the mRNA expression levels of p53, caspase3, CDK 1, 6, 7, 8 and CyclinA1, A2, B1, B2, D1, D2 and D3 were all decreased in the PUF60 knockdown group (Figure 6C-E), which initially indicated that PUF60 was closely related to cell apoptosis and cycle regulation. In the validation of FEN1 and its associated m6A methylation genes, a synergistic expression of FEN 1, Mettl3, Mettle14, WTAP, YTHPF1, YTHPF2, YTHPF3 and PUF60 was also demonstrated (Figure 6F). To explore the downstream molecular events of PUF60, HGC-27 cells with different expression levels of PUF60 were conducted to determine the expression changes of related proteins. Consistent with above results, the experiments of WB further revealed that the expression of p53, bcl-2, ABCA1 and ABCC1 were decreased in PUF60 knockdown cells (Figure 6G). The above positive results greatly suggested that PUF60 may promote chemoresistance in GC through multifactorial forms like drug efflux and apoptosis reduction.

## Discussion

Chemotherapeutic agents such as 5-FU and cisplatin are the main chemotherapy drugs for GC, but the development of drug resistance has become the biggest obstacle affecting clinical efficacy. Therefore, it is crucial to explore chemoresistance biomarkers and related mechanisms. Although previous studies have shown that PUF60 expression is associated with the progression of various cancers^30^, only one report detected the presence of PUF60 secreted protein in GC resistant cell line MGC-803^31^. Notably, it is not clear whether PUF60 plays a direct role in GC chemoresistance.

This study began with the GSE14210 dataset to analyze the gene microarray data from pretreatment and posttreatment endoscopic biopsy samples in GC patients treated with 5-FU and cisplatin. We found that PUF60 was significantly up-regulated after chemotherapy and K-M Plotter analysis showed that higher PUF60 predicted poor prognosis in GC patients treated with 5-FU chemotherapy. This has not been reported before, but this trend is consistent with previous studies that have shown that PUF60 promotes cancer occurrence and development^8^-^10^, ^14^, ^17^. Also, the validation of high PUF60 expression in GC cell lines and tissues is consistent with the existing literature^32^. Based on above results, we focused on revealing the close correlation between PUF60 and GC chemoresistance. We then divided the clinical samples of GC patients into sensitive and non-sensitive groups based on chemotherapy efficacy. As expected, the results of WB, qPCR and IHC experiments showed that the highest expression of PUF60 was demonstrated in the non-sensitive group, especially in the PD group. Here, multiple verifications of clinical samples once again provide strong evidence for PUF60 promoting chemoresistance in GC.

Furthermore, we knocked down PUF60 in the human GC cell line HGC-27 and divided them into control group and knockdown group (sh1 and sh2). We found that the reduction of PUF60 significantly improved the sensitivity and reduced the proliferation capacity of human GC cells when treated with 5-FU or CDDP. Previous studies have also demonstrated that knockdown of PUF60 in either bladder cancer or breast cancer significantly inhibited the proliferation^12^, ^14^ of cancer cells. Moreover, knockdown of PUF60 in HGC-27 cells resulted in increased apoptosis after 5-FU or cisplatin treatment. Therefore, PUF60 promotes chemoresistance to GC *in vitro*, which brings a new point to the field of chemoresistance and the functional role of PUF60 gene itself. One limitation that needs to be addressed is that, though we have conducted CRISPR-cas9 and RNA interference technology in many strains of GC and designed the sequence of more than 20 PUF60 knockdown plasmids, we finally only achieved the knockdown of HGC-27 cells. There is currently no existing literature on the knockdown of PUF60 in gastric cancer, and this limitation may be attributed to the specificity of PUF60. In addition, experimental validation *in vivo* should be conducted.

To explore the underlying mechanism of PUF60 in GC chemoresistance, we conducted series of validation based on enrichment analysis. As is widely known, mechanisms that can initiate or promote direct/indirect drug resistance in human cancer cells include apoptosis inhibition, drug efflux, DNA damage repair, changes in drug targets, and drug inactivation^33^. Here, we integrate the above experimental results and the enrichment function analysis, focusing on the mechanisms of apoptosis and drug efflux. The first was molecules closely related to apoptosis, such as cyclins CDKs, Cyclins, anti-apoptosis protein Bcl-2, and P53; Our experimental results show significant positive results on cell cycle and apoptosis, including the corresponding changes in the mRNA expression levels of p53, CDK 1, 6, 7, 8, CyclinA1, A2, B1, B2, D1, D2, D3. Additionally, we found that the protein expression of p53 and Bcl-2 were decreased in knockdown PUF60 cells. The above positive results consistently illustrate and further explain that PUF60 regulates cell apoptosis through these molecules, thereby promoting chemotherapy resistance in GC. Previous study has also proved that the drug-induced tumor cell death and alteration of the apoptotic pathway is an important mechanism for the development of chemoresistance in cancer cells^34^. Moreover, because the relatively high expression of PUF60 also promotes the chemoresistance of GC to both 5-FU and CDDP, this study has confirmed that the multidrug resistance (MDR) may play a role in this process, including increased drug efflux, inactivation of apoptotic signaling pathway, loss of cell cycle detection point control, improvement of DNA damage repair ability^35^-^37^. Among them, ATP binding cassette transport carrier protein (also known as ABC transporter) plays a particularly critical role in promoting multi-drug resistance to chemotherapy. As expected, the results found that the mRNA and protein levels of ABCA1 and ABCC1 decreased in the PUF60 knockdown group, indicating that PUF60 may promote the efflux of chemotherapeutic drugs through ABCA1 and ABCC1 efflux pumps. Moreover, Early studies have already found a connection between P53 and multidrug transporters, which is contingent upon various factors, including the cellular environment, the drug utilized, and the nature of the p53 mutation^38^. The researchers found a concomitant reduction in ABCC1 mRNA levels with wtp53 expression^38^ and TP53 can affect transcription of ABCC1 by binding to its promoter^39^. There are also results showed that the ERK5/MEF2 pathway controlled ABC expression depending on p53 status^40^. Additionally, reports have shown that p53 regulates ABCA1, with its loss suppressing ABCA1 and activating SREBP1/2 regulators^41^, while also decreasing cholesterol synthetic enzyme transcript levels^42^. Therefore, we hypothesized that on the condition of relatively high expression of PUF60 and the stimulation of chemotherapy drugs like 5-FU and CDDP in GC, p53 is likely to regulate ABCA1 and ABCC1, synergistically promoting chemotherapy resistance in gastric cancer. For sure, a more detailed mechanism is worth further exploration in the future. In short, PUF60 may simultaneously regulate multiple signaling pathways and lead to chemoresistance in GC patients with chemotherapy. Our research results reveal that PUF60 mainly promotes chemoresistance in GC by reducing cell apoptosis and excluding chemotherapy drugs from GC cells through ABCA1, ABCC1.

To our knowledge, this study was the first to uncover a novel function of PUF60 in chemoresistance and prognosis of GC. PUF60 promotes chemoresistance in GC by drug efflux and reducing apoptosis, and it may be adopted as a novel therapeutic target for improving chemotherapy efficacy.

## Supplementary Material

Supplementary figure and table.
