# Supplementary material for: PUF60 Promotes Chemoresistance Through Drug Efflux and Reducing Apoptosis in Gastric Cancer
Source: Int J Med Sci. 2025 Jan 1;22(2):269–82. doi: 10.7150/ijms.102976 (PMC11704696; doi:10.7150/ijms.102976)
Supplement: Supplementary file 1 — Supplementary figure and table. [file ijmsv22p0269s1.zip › Supplementary/Supplementary Figure 1.pdf]

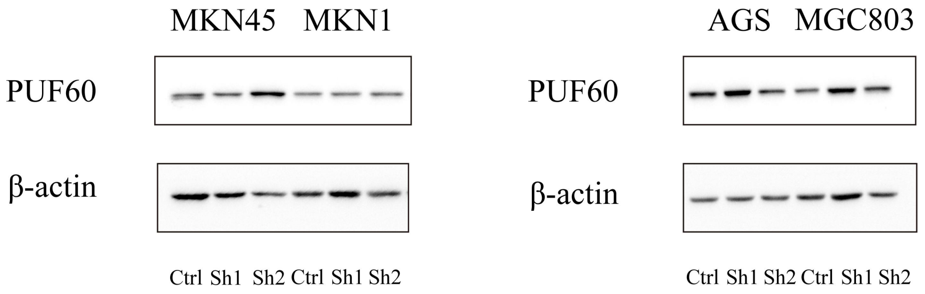

Supplementary Figure 1. Protein validation blots of PUF60 knockdown in gastric cancer cell lines MKN45, MKN1, AGS, and MGC803 (negative results).
